# The Protective Role of Nrf2 in Renal Tubular Cells in Oxidised Low-Density Lipoprotein-Induced Fibrosis

**DOI:** 10.1155/2023/4134928

**Published:** 2023-03-10

**Authors:** Xiangju Long, Zhe Liu, Yanan Sun, Hong Zhang

**Affiliations:** ^1^Nephrology Department, General Hospital of Tianjin Medical University, Tianjin 300052, China; ^2^School of Basic Sciences, Tianjin Medical University, Tianjin 300070, China; ^3^Metabolic Department, Metabolic Hospital of Tianjin Medical University, Tianjin 300000, China

## Abstract

*Background*: CD36 is the receptor of oxidised low-density lipoprotein (OxLDL) in renal tubular epithelial cells. Nuclear factor erythroid 2-related factor 2 (Nrf2) is the key factor in the activation of the Nrf2 signalling pathway and the regulation of oxidative stress. Kelch-like ECH-associated protein 1 (Keap1) is known as an Nrf2 inhibitor. *Methods*: We used OxLDL and Nrf2 inhibitors at different concentrations and durations to treat renal tubular epithelial cells; the expression of CD36 and cytoplasmic and nucleic Nrf2 and E-cadherin in those cells were observed by Western blot and reverse-transcription polymerase chain reaction. *Results*: The protein levels of Nrf2 decreased in expression after 24 hours of OxLDL treatment. At the same time, the Nrf2 protein level in the cytoplasm did not change significantly compared with that of the control group, and the Nrf2 protein level expression in the nucleus increased. Both the messenger ribonucleic acid (mRNA) and protein expression of CD36 decreased following the treatment of cells with the Nrf2 inhibitor Keap1. Kelch-like ECH-associated protein 1 was overexpressed, and CD36 mRNA and protein expression were decreased in OxLDL-treated cells. Following the overexpression of Keap1, E-cadherin expression was reduced in NRK-52E cells. *Conclusion*: Nuclear factor erythroid 2-related factor 2 can be activated by OxLDL; however, it can only alleviate OxLDL-induced oxidative stress by transferring from the cytoplasm to the nucleus. Additionally, Nrf2 may play a protective role by upregulating CD36.

## 1. Introduction

Hyperglycaemia and hyperlipidaemia frequently occur and persist in diabetic nephropathy. Previous studies have shown that abnormal lipid metabolism is an important factor affecting oxidative stress in diabetes. Furthermore, it plays a vital role in the occurrence and development of renal interstitial injury. It has been demonstrated that oxidised low-density lipoprotein (OxLDL) is a major damaging factor in abnormal lipid metabolism [1, 2], and it is also considered an indicator of oxidative stress in serum.

The nuclear factor erythroid 2-related factor 2 (Nrf2) is a major activator of cellular antioxidant response genes. In normal cells without oxidative stress, Nrf2 is maintained at low levels in the cytoplasm by its inhibitor Kelch-like ECH-associated protein 1 (Keap1) for proteasomal degradation. Under oxidative and other stress, the ubiquitin ligase activity of Keap1 is inactivated, allowing Nrf2 to accumulate in the nucleus and bind as a heterodimer. Additionally, small Minor Allele Frequency (MAF) proteins bind to the antioxidant response elements of target genes, such as *NQO1*, *HO1*, and *GCLM*, stimulating their expression [3, 4].

Previous studies have shown that OxLDL plays a key role in the pathogenesis of atherosclerosis and can regulate cell growth, the expression of transcription factors and cytokines, and affect cell viability [5, 6]. Previous cytological studies have found that OxLDL in preadipocytes is a specific saturation process mediated by CD36, which affects adipose tissue homeostasis by inhibiting the differentiation of preadipocytes [7]. Oxidised low-density lipoprotein has also been shown to induce CD36 overexpression via the upregulation of Nrf2 expression in preadipocytes. In adipocytes, the upregulation of CD36 may indicate a compensatory mechanism to meet the requirement for excessive OxLDL and oxidised lipids in blood and reduce the risk of atherosclerosis [8]. However, whether the same mechanism exists in renal tubular cells remains unclear. In addition, it is unclear how abnormal lipid metabolism leads to oxidative stress and, ultimately, renal interstitial injury. This study found that Nrf2 could be activated by OxLDL and that Nrf2 may play a protective role by upregulating CD36 expression.

## 2. Materials and Methods

### 2.1. Cell Culture

*Rattus norvegicus* renal tubular epithelial cell line NRK-52E (American Type Culture Collection [ATCC], VA, USA) and human renal tubular epithelial cell line HK-2 (ATCC) were plated and cultured in Dulbecco's modified Eagle's medium supplemented with 10% foetal bovine serum. HEK293T cells were obtained from the ATCC and used for *Lentivirus* transfection.

### 2.2. OxLDL Preparation and Treatment

Oxidised low-density lipoprotein was obtained from Yiyuan Biotechnology Company (Guangzhou, China) and sterilised using a 0.22 *μ*m membrane (Millipore Corp., Bedford, MA, USA). Different concentrations of OxLDL were tested. First, 50 mg/L OxLDL was incubated with NRK-52E cells for 0–48 hours. Then, 0, 100, and 150 mg/L concentrations of OxLDL were incubated with the cells for 0–4 days. In some experiments, NRK-52E cells infected with empty p305 and p305-Keap1 were treated with 150 mg/L OxLDL for 2 or 3 days. Cells were treated to express CD36, total Nrf2, nuclear Nrf2, NF*κ*B, and E-cadherin at the end of the incubation period as described.

### 2.3. Serum Starvation

Cells were seeded in culture dishes, first cultured with 10% foetal bovine serum, followed by cell starvation with 1% foetal bovine serum 24 hours later. Intracellular proteins were collected for subsequent experiments after cell starvation for 48 hours.

### 2.4. Immunoblotting Analysis

#### 2.4.1. Nuclear and Cytosolic Protein Extracts

The NRK-52E and HK-2 cells infected with empty p305 and p305-Keap1 were washed with ice-cold phosphate-buffered saline (PBS) and lysed in an appropriate volume (10 × cell volume) of ice-cold buffer A containing 0.5% NP-40. After incubation, the cells were washed with PBS and lysed in a lysis buffer (Bio-Rad Laboratories Inc., Hercules, CA, USA). The cell lysates were sonicated and centrifuged at 13,000 rpm at 4°C for 15 minutes to pellet the cell debris. Before Western blotting, labelled dye and 2-mercaptoethanol were added to the lysate, separated by sodium dodecyl sulphate–polyacrylamide gel electrophoresis and transferred to a nitrocellulose membrane (Pall Corp., AZ, USA). After being blockaded with 5% non-fat milk, the membranes were incubated at 4°C overnight with rabbit polyclonal anti-CD36 antibody (Abcam, Cambridge, UK), rabbit polyclonal anti-Nrf2 antibody (Abcam, Cambridge, UK), and mouse mAb to E-cadherin (BD Biosciences, San Jose, CA, USA) or rabbit polyclonal to NF*κ*B (Santa Cruz Biotechnology, Inc., Dallas, TX, USA). After extensive washing in Tris-buffered saline (TBS)–Tween 20 (TBST), the membranes were incubated with horseradish peroxidase-conjugated anti-mouse immunoglobulin G (IgG) or anti-rabbit IgG (Zhongshan Golden Bridge Biotechnology Co. Ltd., Beijing, China) for 1 hour at room temperature. After washing with TBST, the membranes were incubated with an enhanced chemiluminescence system detection kit (Millipore Corp., Bedford, MA, USA).

1 mM dichlorodiphenyltrichloroethane (DTT) and 1 × protease inhibitor (PI). The cell lysates were pipetted up and down several times to disrupt any cell clumps, rotated for 10 minutes at 4°C, and centrifuged at high speed for 3 minutes at 4°C. The supernatants (cytosolic part) were stored at 4°C. The pellets were washed with ice-cold buffer A and mixed with 3 × volumes of ice-cold buffer B containing 1 DTT and 1 × PI. The mixtures were incubated on ice for 30 minutes with an intermittent strong vortex and spun for 15 minutes at high speed at 4°C. The supernatants were collected, and the concentration of proteins was determined using a bicinchoninic assay.

#### 2.4.2. Total Ribonucleic Acid Extraction and Reverse-Transcription Polymerase Chain Reaction Analysis

Total ribonucleic acid (RNA) was isolated from the NRK-52E cells, the NRK-52E cells infected with empty p305 and p305-Keap1, and the OxLDL-treated cells using TRIzol as a reagent (Invitrogen Life Technologies, Thermo Fisher Scientific, Inc., MA, USA). Quantitative real-time polymerase chain reaction (PCR) was performed with primers using a sequence detector (Applied Biosystems, Thermo Fisher Scientific, Inc., MA, USA). Oligonucleotide primers used for the reverse-transcription (RT) PCR were as follows: 5′;-GGTGTGCTCAACAGCCTTATC-3′; and 5′;-TTATGGCAACCTTGCTTATG-3′; for detecting rat CD36 messenger RNA (mRNA), 5′;-GACTGGATCTGGCATAAAGA-3′;, and 5′;-TCAACGGCACAGTCAAGG-3′; and 5′;-ACTCCACGACATACTCAGC-3′; for rat glyceraldehyde-3-phosphate dehydrogenase (GAPDH) mRNA. The expressions of the *CD36* and *LOX-1* genes were determined as the amount of CD36 relative to GAPDH mRNA using the comparative C_T_ method described in the Applied Biosystems (ABI) sequence detection system.

#### 2.4.3. Lentivirus Transfection

*Lentivirus* was produced in HEK293T cells packed with plasmid pMD2 using plasmids p305 and p305-Keap1. BSBG, pMDLg/pRRE, and pRSV-REV were obtained using a Ca_3_(PO_4_)_2_ transfection kit (Millipore Corp., Bedford, MA, USA). NRK-52E cells were infected with a *Lentivirus* vector until the Enhanced Green Fluorescent Protein (EGFP) marker was completely displayed as successfully infected cells.

## 3. Results

### 3.1. Expression of CD36 in NRK-52E Cells

**Figure 1:**
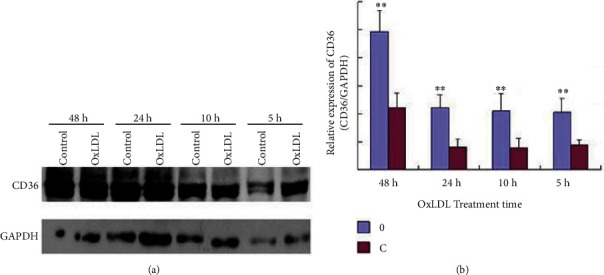
(a) CD36 expression in NRK-52E cells incubated by 50 mg/L OxLDL (C: control). The expression of CD36 in NRK-52E cells incubated by 50 mg/L OxLDL, OxLDLD was made to different concentration, and incubated NRK-52E cells for different time 0, 5, 10, 24, and 48 hours. Collected all groups of cells and test CD36 level by Western-blotting, the results show the CD36 level of 5, 10, 24, and 48 hours groups increased (*p* < 0.01); and CD36 level of 24 and 48 hours groups more apparently. (b) CD36 expression in NRK-52E cells with 50 mg/L OxLDL treatment. (O: OxLDL; C: control; ∗∗: *p* < 0.01).

NRK-52E cells were incubated with 50 mg/L OxLDL for 0, 5, 10, 24, and 48 hours, respectively. The cells were collected, and the expression of CD36 was detected by Western blot. The results showed that CD36 expression was reduced under OxLDL-treated conditions (Figures 1(a) and 1(b)).

**Figure 2:**
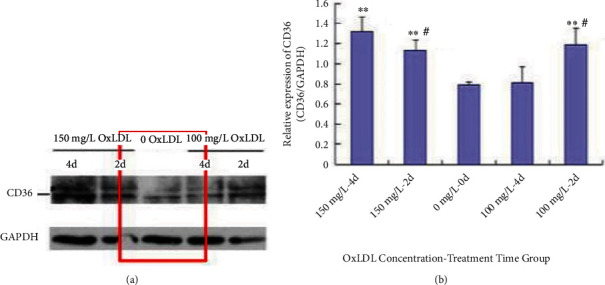
(a) Expression of CD36 after treatment of NRK-52E cells with different OxLDL concentrations. (b) Expression of CD36 after treatment of NRK-52E cells with different OxLDL concentrations (∗∗: *p* < 0.01; #: 2 days compared with the 4-day group).

Different concentrations of OxLDL were prepared with cell culture media (0, 100, and 150 mg/L) and incubated with NRK-52E cells under different concentrations of OxLDL for 2 and 4 days. The results showed that the expression of the CD36 protein in the NRK-52E cells stimulated by 150 mg/L OxLDL was higher than that in the 0 mg/L OxLDL group on days 2 and 4 (*p* < 0.05); the 4-day group was more evident than the 2-day group. When NRK-52E cells were stimulated with 100 mg/L OxLDL for 2 days, the expression of the CD36 protein in the 100 mg/L group also increased (*p* < 0.05), but the growth range was relatively small compared with that of the 150 mg/L group (*p* < 0.05). After 4 days, the expression of the CD36 protein in the NRK-52E cells stimulated by 100 and 150 mg/L OxLDL was not statistically significant (*p* > 0.05) (Figures 2(a) and 2(b)).

**Figure 3:**
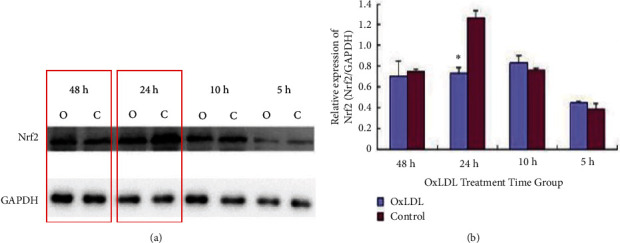
(a) Total Nrf2 protein levels detected in NRK-52E cells with 50 mg/L OxLDL treatment (C: control; O: OxLDL processing). (b) Total Nrf2 protein levels detected in NRK-52E cells with 50 mg/L OxLDL treatment (note: C: control; O: OxLDL treatment; ∗Compared with the control group, *p* < 0.05).

Changes in Nrf2 protein levels under OxLDL treatment were determined. Oxidised low-density lipoprotein was prepared to a concentration of 50 mg/L using a cell culture medium; subsequently, NRK-52E cells were stimulated for 0, 5, 10, 24, and 48 hours. The cells were then collected, and the total Nrf2 protein level in the cells was detected. The results showed no significant change in the total Nrf2 protein level in the NRK-52E cells at 5 and 10 hours (*p* > 0.05). After 24 hours of treatment, the results revealed a reduced expression of Nrf2 compared with that of the control group (*p* < 0.05) and a reduced expression with no statistical significance after 48 hours (*p* > 0.05) (Figures 3(a) and 3(b)).

**Figure 4:**
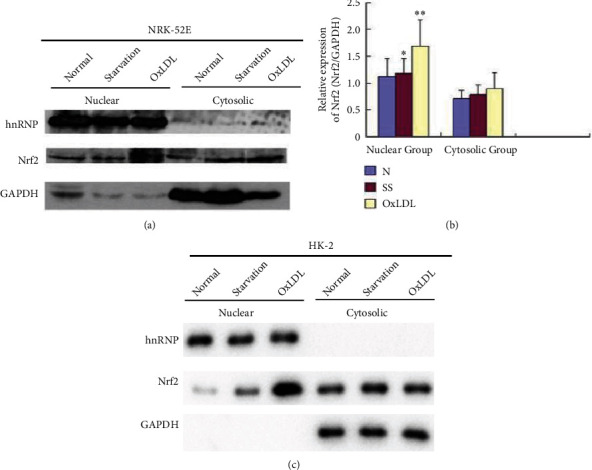
(a) Nrf2 protein expression in nucleus and cytoplasm of NRK-52E cells treated with 150 mg/L OxLDL and serum starvation (SS: serum starvation). hnRNP was used as a marker for nuclear. GAPDH was used as a marker for cytoplasm. (b) Levels of Nrf2 nuclear protein and cytosolic protein were detected after 150 mg/L OxLDL and serum starvation treatment (N: normal control group; SS: serum starvation group; OxLDL: OxLDL treatment group; ∗Compared with the control group, *p* < 0.05; ∗∗Compared with the control group, *p* < 0.01). (c) Nrf2 protein expression in nucleus and cytoplasm of HK-2 cells treated with 150 mg/L OxLDL and serum starvation (SS: serum starvation). hnRNP was used as a marker for nuclear. GAPDH was used as a marker for cytoplasm.

NRK-52E cells were treated with 150 mg/L OxLDL, and the nuclear and cytoplasmic proteins were extracted to detect the Nrf2 protein level. The results showed that the Nrf2 protein in the NRK-52E nucleus of the serum starvation and OxLDL treatment groups was higher than that of the control group (*p* < 0.05). In contrast, the Nrf2 protein quantity in the cytoplasm of the serum starvation and OxLDL treatment groups was not different (*p* > 0.05) (Figures 4(a) and 4(b)). We obtained the same results in human renal tubular epithelial cell line HK-2 cells (Figure 4(c)).

### 3.2. Keap1 and Nrf2 Effects on CD36 Expression

**Figure 5:**
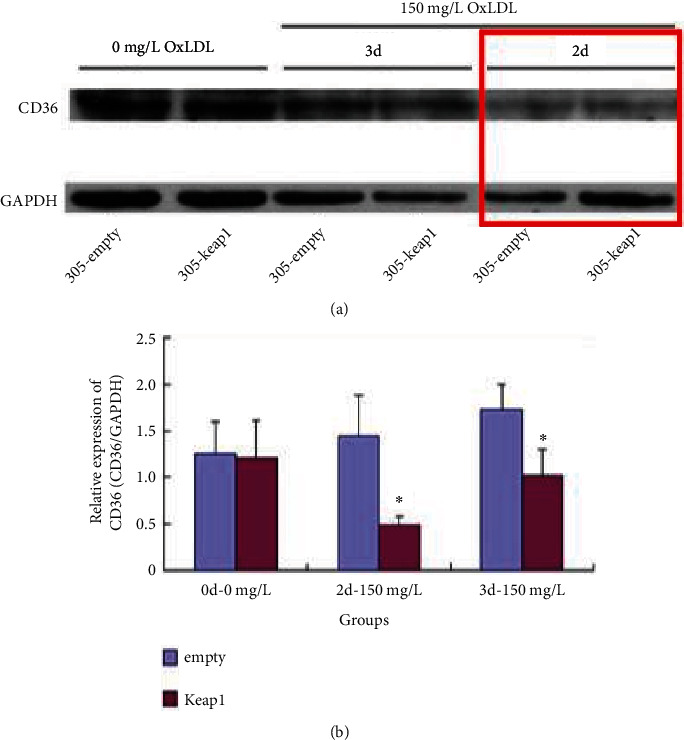
(a) CD36 expression in NRK-52E cells after Keap1 over overexpression and OxLDL treatment. (b) CD36 expression in NRK-52E cells after Keap1 overexpression and OxLDL treatment (note: ∗compared with the control group, *p* < 0.05).

Keap1 is an inhibitory protein of Nrf2. NRK-52E cells were treated with Keap1 overexpression (the p305-Keap1 group) and were untreated in the control group (p305-empty group); then, the NRK-52E cells were stimulated with 0 and 150 mg/L OxLDL for 2 and 3 days, respectively. The results showed that when stimulated with 0 mg/L OxLDL, there was no change in the expression of CD36 in the NRK-52E cells treated with Nrf2 inhibitor Keap1 overexpression (the p305-Keap1 group) compared with that of the untreated control group (the p305-empty group). Compared with the untreated control NRK-52E cell group, the expression of CD36 in the Keap1-overexpressed NRK-52E cell group decreased after 2 and 3 days of stimulation with 150 mg/L OxLDL (*p* < 0.05) (Figures 5(a) and 5(b)).

**Figure 6:**
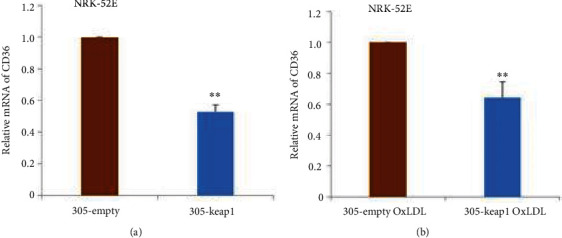
(a) Comparison of a treatment group (305-keap1 group) with untreated control group (305-empty group) (Note: ∗∗Compared with the control group, *p* < 0.01). (b) Comparison of CD36-mRNA in Nrf2 inhibitor 305-keap1 transfection OxLDL treatment group (305-keap1-OxLDL group) with 305-keap1 untreated control group (305-empty OxLDL group) (note: ∗∗compared with the control group, *p* < 0.01).

To determine whether Nrf2 activation is related to OxLDL-mediated renal tubular cells, we treated renal tubular cells with the Nrf2 inhibitor Keap1 (the p305-Keap1 group), and the control group (the p305-empty group) was not treated. Then, both groups of cells were stimulated with OxLDL (the p305-Keap1-OxLDL group). The results showed that the CD36 mRNA in the Keap1 group was lower than that in the control group, and the Keap1 OxLDL group also showed a significant decrease in CD36 mRNA compared with that of the control group (Figures 6(a) and 6(b)).

### 3.3. E-Cadherin Protein Expression in Tubular Cells Treated with Keap1

**Figure 7:**
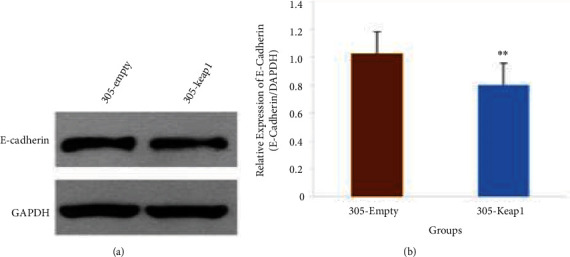
(a) E-cadherin expression in 305-keap1 NRK-52E cells group compared with control group cells. (b) E-cadherin expression in 305-keap1 NRK-52E group compared with control group cells (note: ∗∗compared with the control group, *p* < 0.01).

To determine the relationship between Nrf2 and renal tubular fibrosis, we treated renal tubular cells with the Nrf2 inhibitor p305-Keap1 (the p305-Keap1 group). Then, we detected the expression of fibrosis factor E-cadherin in the treatment and control groups. The results showed that the expression of E-cadherin in the p305-Keap1 group was lower than that of the control group (Figures 7(a) and 7(b)).

## 4. Discussion

This study found that the CD36 protein, an OxLDL membrane receptor, increased in OxLDL-treated renal tubular NRK-52E cells. The results revealed that OxLDL could upregulate CD36, which was related to the concentration and action time of OxLDL. The upregulation effect of 150 mg/L OxLDL on CD36 was more evident than with 50 and 100 mg/L OxLDL. The expression level of the CD36 protein increased with a prolongation of action time and an increase in OxLDL concentration. The CD36 protein changed the 24-hour OxLDL intervention by at least 50 mg/L, indicating that the progress of OxLDL-mediated renal tubular injury was slow. This study confirmed for the first time that the OxLDL receptor CD36 in renal tubular cells plays a role as an OxLDL receptor during hyperlipidaemia and oxidative stress. The upregulation of CD36 indicates the coping mechanism after excessive OxLDL in hyperlipidaemia to reduce the risk of renal tubular injury caused by OxLDL.

Additionally, we found that in a high OxLDL environment, CD36 was upregulated, and Nrf2 was changed. After treatment with 50 mg/L OxLDL for 5 and 10 hours, the total Nrf2 protein level did not change significantly, although it decreased after treatment for 24 and 48 hours. These results indicate that the OxLDL-mediated Nrf2 pathway needs sufficient time to function, which is consistent with the results of previous studies on preadipocytes [9–12].

Furthermore, the present study showed that NRK-52E cells treated with OxLDL and starvation for 2 days had a relatively high level of Nrf2 protein in the nucleus and a relatively low level in the cytoplasm; this indicated that OxLDL stimulated the initiation of oxidative stress and activated the Nrf2 signalling pathway by causing the transfer of Nrf2 from the cytoplasm to the nucleus, continuing to regulate the downstream gene *HO-1* [8]. This study may confirm the regulatory mechanism of Nrf2 in renal tubular cells under oxidative stress and abnormal lipid metabolism. The relationship between the levels of CD36 and Nrf2 proteins in renal tubular cells after OxLDL treatment may indicate that OxLDL initiates the Nrf2 protective mechanism by upregulating CD36.

The cytoplasmic inhibitor Keap1 is a receptor that induces Nrf2 activation via oxides and electrophiles. It is also a regulator of Nrf2 degradation mediated by the ubiquitin protease system and plays a central role in regulating the Nrf2 signalling pathway [11, 12]. Keap1 has been shown to be a shuttle protein that can move back and forth between the cytoplasm and nucleus. Once intracellular redox homeostasis is restored, Keap1 can shuttle from the cytoplasm into the nucleus and move Nrf2 upwards from antioxidant response elements; then, the Nrf2–Keap1 complex moves out of the nucleus again. The Nrf2–Keap1 complex is again degraded in the cytoplasm by a cul3-rbxl-e3-dependent ubiquitinase mechanism. Nuclear factor erythroid 2-related factor 2 remains at low-level expression, and the Nrf2 signalling pathway is inactivated [13, 14]. Therefore, Keap1 can be considered to play a role in inhibiting the transfer of Nrf2 to the nucleus [15–17].

This study showed that the expression of CD36 mRNA and protein decreased in NRK-52E renal tubular cells treated with Keap1. After OxLDL stimulation and Keap1 overexpression treatment, the expression of CD36 mRNA and protein in renal tubular cells also decreased. These results suggest that Nrf2 plays a role in regulating OxLDL receptor CD36 and activates the protective mechanism of oxidative stress by upregulating CD36.

Strutz [18] first proposed the concept of renal tubular phenotypic transformation (epithelial–mesenchymal transition [EMT]) in a renal tubular basement membrane cell model, which can be transformed into medial forebrain bundle cells and express the interstitial marker vimentin [18]. The main feature of renal tubular cells after EMT is the reduction or loss of E-cadherin expression [19]. Renal tubular epithelial cells maintain the integrity of cell morphology, structure, and function through various cell adhesion mechanisms, such as E-cadherin. The loss of E-cadherin leads to the transformation of the characteristics of primary epithelial cells to non-epithelial functions [20]. Therefore, E-cadherin is the main molecular marker of epithelial adhesion and phenotype and can be used as an important marker of EMT. The decreased expression of E-cadherin can be used as a marker of renal tubular EMT and fibrosis. Therefore, in this study, we selected E-cadherin as a molecular marker of OxLDL-induced renal tubular cell fibrosis [20, 21]. Our results showed that the expression of E-cadherin decreased in renal tubular cells treated with Keap1. This result may indicate that Nrf2 plays an inhibitory role in the EMT process of renal tubular cells. Moreover, these results confirmed that Nrf2 has an inhibitory effect on the process of renal tubulointerstitial fibrosis and plays a protective role in renal tubulointerstitial fibrosis. The protective effect of Nrf2 is finally reflected in the inhibition of the EMT response by reducing the loss of E-cadherin and inhibiting the fibrosis process in renal tubular cells.

## 5. Conclusions

Overall, this study's results suggest that Nrf2 plays a key role as a transcriptional regulator of oxidative stress in OxLDL-induced renal fibrosis. Excessive OxLDL in renal tubular cells upregulates CD36 and activates the Nrf2 signalling pathway, resulting in the transfer of Nrf2 from the cytoplasm to the nucleus; this may regulate CD36 at a later stage, leading to upregulation of CD36, thereby enhancing CD36's ability to defend against OxLDL and play a protective role. E-cadherin is worthy of further study and discussion regarding reducing renal tubular cell injury and delaying nephropathy. Research should focus on future therapeutic targets.

## Data Availability

The generated or analyzed data used to support the findings of this study are included within the article.

## References

[1] Singh D. K., Winocour P., Farrington K. (2011). Oxidative stress in early diabetic nephropathy: fueling the fire. *Nature Reviews. Endocrinology*.

[2] Pedzik A., Paradowski M., Rysz J. (2010). Oxidative stress in nephrology. *Polski Merkuriusz Lekarski*.

[3] Rojo de la Vega M., Chapman E., Zhang D. D. (2018). NRF2 and the hallmarks of cancer. *Cancer Cell*.

[4] Ge W., Zhao K., Wang X. (2017). iASPP is an antioxidative factor and drives cancer growth and drug resistance by competing with Nrf2 for Keap1 binding. *Cancer Cell*.

[5] Myara I., Alamowitch C., Michel O. (2003). Lipoprotein oxidation and plasma vitamin E in nondiabetic normotensive obese patients. *Obesity Research*.

[6] Toshima S., Hasegawa A., Kurabayashi M. (2000). Circulating oxidized low density lipoprotein levels. *Arteriosclerosis, Thrombosis, and Vascular Biology*.

[7] Viora M., Straface E., Di G. G. (1997). Oxidized low density lipoproteins impair peripheral blood mononuclear cell proliferation and cytokine production. *Biochemical and Biophysical Research Communications*.

[8] D’Archivio M., Scazzocchio B., Filesi C. (2008). Oxidised LDL up-regulate CD36 expression by the Nrf2 pathway in 3T3-L1 preadipocytes. *FEBS Letters*.

[9] Moi P., Chan K., Asunis I., Cao A., Kan Y. W. (1994). Isolation of NF-E2-related factor 2 (Nrf2), a NF-E2-like basic leucine zipper transcriptional activator that binds to the tandem NF-E2/AP1 repeat of the beta-globin locus control region. *Proceedings of the National Academy of Sciences of the United States of America*.

[10] Sykiotis G. P., Habeos I. G., Samuelson A. V., Bohmann D. (2011). The role of the antioxidant and longevity-promoting Nrf2 pathway in metabolic regulation. *Current Opinion in Clinical Nutrition and Metabolic Care*.

[11] Ryoo I. G., Ha H., Kwak M. K. (2014). Inhibitory role of the KEAP1-NRF2 pathway in TGF*β*1-stimulated renal epithelial transition to fibroblastic cells: a modulatory effect on SMAD signaling. *PLoS One*.

[12] Miyata T., Suzuki N., van Ypersele de Strihou C. (2013). Diabetic nephropathy: are there new and potentially promising therapies targeting oxygen biology?. *Kidney International*.

[13] Saito H. (2013). Toxico-pharmacological perspective of the Nrf2-Keap1 defense system against oxidative stress in kidney diseases. *Biochemical Pharmacology*.

[14] Kim H. J., Vaziri N. D. (2010). Contribution of impaired Nrf2-Keap1 pathway to oxidative stress and inflammation in chronic renal failure. *American Journal of Physiology. Renal Physiology*.

[15] Itoh K., Wakabayashi N., Katoh Y., Ishii T., O’Connor T., Yamamoto M. (2003). Keap1 regulates both cytoplasmic-nuclear shuttling and degradation of Nrf2 in response to electrophiles. *Genes to Cells*.

[16] Ha H., Lee H. B. (2000). Reactive oxygen species as glucose signaling molecules in mesangial cells cultured under high glucose. *Kidney International. Supplement*.

[17] Lan H. Y. (2012). Transforming growth factor-*β*/Smad signalling in diabetic nephropathy. *Clinical and Experimental Pharmacology & Physiology*.

[18] Strutz F. M. (2009). EMT and proteinuria as progression factors. *Kidney International*.

[19] Lan H. Y. (2011). Diverse roles of TGF-*β*/Smads in renal fibrosis and inflammation. *International Journal of Biological Sciences*.

[20] Tian X., Liu Z., Niu B. (2011). E-cadherin/*β*-catenin complex and the epithelial barrier. *Journal of Biomedicine & Biotechnology*.

[21] Lee J. R., Muthukumar T., Dadhania D. (2014). Urinary cell mRNA profiles predictive of human kidney allograft status. *Immunological Reviews*.

